# Quality Control System of Red Jujube by Hybrid Model: Development of an Efficient Framework

**DOI:** 10.3389/fpls.2022.888978

**Published:** 2022-06-09

**Authors:** Rongzhi Jing, Ping Li

**Affiliations:** School of Electronic and Information Engineering, Sias University, Xinzheng, China

**Keywords:** red jujube quality and safety, traceability system, blockchain, internet of things (IoTs), quality control system

## Abstract

Food traceability is very important for the quality and safety of agricultural products, which is related to the people’s livelihood and national economy and has drawn great attention from governments and scientists around the world. The existing studies have not yet overcome the crisis characteristics comprehensively and systematically. A traceability system of red jujube is constructed by a hybrid mode of blockchain and the Internet of Things (IoTs). The system integrates the blockchain and the IoT technologies with characteristics of tamper-proof, decentralization, and distributed storage and solves the problem of date quality traceability by designing the technical process and architecture of date quality traceability and the big data of red jujube, jujube plantation, processing enterprise, commercial enterprises, and market administration. The whole process from planting to processing and sales of red jujube are recorded in the block to ensure the realization of quality traceability of red dates in the process. Through the whole process of big data processing, the key information collected in each process is stored in the database to ensure the realization of quality traceability of red dates in the framework. The system can help to minimize the production and distribution of unsafe or poor-quality products, thereby minimizing the potential for bad publicity, liability, and recalls.

## Introduction

The quality and safety of agricultural products are related to the people’s livelihood and national economy. The frequent occurrence of agricultural product quality and safety crises has drawn great attention from countries around the world. Quality control in agricultural production and throughout the supply chain is one of the most challenging issues in the world today, especially when it comes to typical food products. The quality and safety of agricultural products are an important study direction at present, which is a multidisciplinary study field. Its purpose is to explore the technology, measure and supervision guarantee system to ensure the hygiene and food safety of agricultural products, reduce the potential disease, and prevent the risk of food poisoning in the process of agricultural products processing, storage, and sales. In recent years, many agricultural quality control systems have been presented and various companies are trying to experiment with joint use of radiofrequency identification (RFID), Internet of Things (IoTs), and blockchain technologies to solve problems in scenarios where numerous untrusted actors get involved (Galvez et al., 2018; Corallo et al., 2020).

Good traceability systems help to minimize the production and distribution of unsafe or poor quality products and minimize the potential for bad publicity, liability, and recalls. The current food labeling system cannot guarantee that the food is authentic, good quality, and safe. Zhao et al. (2019) presented comprehensive information about traceability with regard to safety and quality in the food supply chain and applied as a tool to assist in the assurance of food safety and quality, as well as to achieve consumer confidence.

Blockchain technology is a new digital technological approach underpinned by the Industry 4.0. It is used to ensure data integrity and prevent tampering and single point of failure through offering fault tolerance, immutability, trust, transparency, and full traceability of the stored transaction records to all the agri-food value chain partners. Zhao et al. (2019) used systematic literature network analysis to review the state-of-the-art blockchain technology, including its recent advances, main applications in agrifood value chain, and challenges from a holistic perspective. They identified six challenges that have been included storage capacity and scalability, privacy leakage, high cost and regulation problem, throughput and latency issue, and lack of skills. Yadav et al. (2020) identified seven enablers by grouping thirty subenablers, developed the Internet of Things (IoTs)-based efficient and supportive coordinating system for enhancing the coordinating mechanism in Agriculture Supply Chain Management (ASCM) under natural outbreaks and discussed a case study of the sugar mill industry. Zu et al. (2020) analyzed and researched in detail the typical crisis events of agricultural product quality and safety in China from 2004 to 2018. They extracted 13 abstract features to form a set of agricultural product quality and safety crisis features. Their studies are helpful to enrich the study results of quality and safety management of agricultural products in China and take effective measures to reduce the harm. Rakitskii et al. (2020) quantified 42 active ingredients of pesticides (and their metabolites) in samples of rice grain, dragon fruit (pitahaya), avocado, mango, and banana (fresh and dried) and used the sample preparation procedure with QuEChERS technology for the multiresidues determination of the 40 compounds. The results show the safety of certain types of food products imported from Vietnam by the content of residual quantities of pesticides. Demestichas et al. (2020) overviewed the application of blockchain technologies for enabling traceability in the agri-food domain and presented definitions, levels of adoption, tools, and advantages of traceability, accompanied with a brief overview of the functionality and advantages of blockchain technology. They conducted an extensive literature review on the integration of blockchain into traceability systems and discussed the relevant existing commercial applications, highlighting the relevant challenges and future prospects of the application of blockchain technologies in the agri-food supply chain. Feng et al. (2020) reviewed the blockchain technology characteristics and functionalities, provided valuable information for researchers and practitioners on the use of blockchain-based food traceability management and proposed an architecture design framework and suitability application analysis flowchart of blockchain-based food traceability systems. By combining smart contracts, Interplanetary File System, and the Internet of Things, Cocco and Mannaro (2021) tried to address these issues and presented a proposal of an implementation model for the supply chain management of a typical Italian Carasau bread. The method can guarantee and certify a transparent, secure, and auditable traceability in such a way that each actor of the supply chain can verify the quality of the product. Ray et al. (2021) looked into manufacturer–retailer collaboration in the UK food supply chain and aimed to develop a preliminary conceptual framework by identifying the key factors that influence long-term performance and accuracy of collaborative forecasting and developed a new model for Synchronized Information Forecast Collaboration (SIFC). Zhao and Ning (2017) constructed an agricultural traceability system of agricultural products through the IoT technologies of information, security, and cloud computing. This system can provide effective basis for agricultural trade, logistics, and safe consumption. The customers can effectively understand the detailed process and risk status of every phase of agricultural products through this system. Almalki et al. (2021) presented a low-cost platform for comprehensive environmental parameter monitoring using the IoT to help farmers, government, or manufacturers to predict environmental data over the geographically large farm field, which can enhance crop productivity and farm management in a cost-effective and timely manner. Yang and Sun (2020) introduced blockchain technology and proposed a blockchain-based data management system to afford efficient data extraction, management, and access control for heterogeneous forms of data across the agricultural supply chain. Zhang et al. (2020) analyzed the operation mechanism and development path of agricultural product supply chain by blockchain technology and reconstructed the agricultural product supply chain based on the advantages of blockchain technology. Blockchain makes data public for all the drones and enables drones to log information concerning world states, time, location, resources, delivery data, and drone relation to all the neighbor drones. Alsamhi et al. (2021) introduced decentralized independent multidrones to accomplish the task collaboratively and discussed end-to-end delivery application of combination of blockchain and multidrone in combating coronavirus disease 2019 (COVID-19) and beyond future pandemics. Edge computing has prospects in agricultural applications, such as safety traceability of agricultural products, pest identification, unmanned agricultural machinery, and intelligent management. It is possible to apply federated learning to beyond 5G by development of edge computing makes. Alsamhi et al. (2022) proposed a blockchain empowered federated learning framework, presented its potential application scenarios in beyond 5G, and designed a deep reinforcement learning-based algorithm to find an optimal solution to the problem. The results showed that the proposed scheme is effective. Zhang et al. (2021) primarily reviewed the application of edge computing in the agricultural IoTs and investigated the combination of edge computing and artificial intelligence, blockchain, and virtual/augmented reality technology. Tharatipyakul and Pongnumkul (2021) gathered 25 review articles on blockchain or agri-food supply chain and 39 study articles that presented screenshots of user interfaces of related applications, reviewed 7 review articles that focused on the blockchain-based agri-food supply chain to understand the benefits and challenges in the blockchain applications, aimed to address this gap by reviewing existing works from user interface perspectives, and analyzed 14 blockchain-based agri-food traceability applications and 10 non-blockchain-based agri-food traceability applications. Finally, they discussed the study gaps and future study directions related to user interface design, which should be addressed to ease future blockchain adoption.

Agricultural products traceability management integrated system can produce the data uploaded to the agricultural products of the whole process of cultivation of traceability management integrated system, the digital information, so supervision department can do random inspection of agricultural products, through the data to verify reliability, record results, give consumers a greater sense of trust, enhance consumer confidence. The problems of traceability agricultural products system are summarized as: (1) Enterprises developing traceability agricultural products system are not the same, so traceability information cannot be shared, many system software are not compatible, and the purpose of query cannot be achieved in different systems. Some query terminals can only be queried in supermarkets, which make it inconvenient to use; (2) At present, the traceability system is not unified in many aspects, such as identification code, storage information, and network query system, and it also faces different kinds of food; and (3) The accuracy of traceability is not high, some of them can only be traced back to the enterprise, and the specific process of planting, processing, or transportation cannot be traced.

Xinzheng red jujube (XRJ) is a specialty of Xinzheng city, Zhengzhou City, Henan Province, China. It is a good fruit nourishing blood and spleen beauty, has high medicinal value, and its leaves, flowers, fruits, skins, roots, and thorns can be used as medicine. However, the development of XRJ industry is also facing many problems, such as the quality and safety system is not perfect, especially the pesticide and fertilizer residues are too high, which greatly restrict the development pace of XRJ industry. The quality and safety of XRJ is not only a major livelihood issue related to people’s health, but also a major obstacle to the international XRJ trade. To guarantee safety in food, an efficient tracking and tracing system is required. RFID devices allow recording all the useful information for traceability directly on the commodity. By analyzing the problems existing in the quality and safety of XRJ and discussing the quality and safety problems of XRJ based on the IoT, this article tries to construct a quality control system of XRJ based on the IoT, improve the quality and safety control level of XRJ, and then realize the intelligent and scientific management of XRJ production, which have important practical significance for promoting the development of modern XRJ. This proposed system aims to reduce the chemical pesticides, fertilizers and antibiotics usage, stimulating sustainable food consumption, and promoting affordable healthy food for all. Consumers are encouraged to choose healthy and sustainable diets and reduce food waste. Farmers and producers are encouraged to provide more details about food origin, nutritional value, and environmental footprint. The contributions of this article are as follows.

(1) A hybrid mode of blockchain and the IoT is used to construct a quality control system of XRJ.
(2) Data processing of XRJ traceability system is introduced in detail.
(3) The XRJ traceability system is verified in the Henan Xinzheng Xinxing Jujube Industry Corporation Ltd.

The rest of the article is organized as follows. In Section 2, the related work is introduced. Section 3 focuses on XRJ quality traceability system in detail. The experiments and analysis are conducted in Section 4. Section 5 concludes the study work and point out the future work.

## Related Work

### Agricultural Internet of Things

The Internet of Things (IoTs) is an extended network based on the internet through a variety of sensors, RFID technology, global positioning system (GPS), infrared sensors, laser scanner, and other equipment and technology, no need to monitor real-time acquisition, connected, interactive object or process, collect the sound, light, heat, electricity, mechanics, chemistry, biology, location, and other needed information, through all the kinds of possible internet access, to realize the ubiquitous connection between objects and objects, objects and people, and realize the intelligent perception, recognition, and management of objects and processes. The Internet of Things is an information carrier based on the internet, traditional telecommunications networks, etc. It enables all the ordinary physical objects that can be independently addressed to form an interconnected network (Mukkamala et al., 2018).

At present, XRJ production mainly depends on manual experience management and lack of scientific and systematic guidance. Information acquisition is one of the most important key technologies to realize the high level of facility production of XRJ and to optimize the facility biological environment control. The agricultural IoT plays a crucial role in revolutionizing agricultural production. From the practical experience of developed countries, the IoT has many applications in the field of agriculture and is developing in the direction of reliability, energy saving, environmental adaptability, low cost, information, and intelligence. If it is a jujube production base of thousands of acres, if the IoT technology is applied, manual control only needs to click the mouse tiny action, only a few seconds before and after, can completely replace the tedious manual operation such as watering and fertilization. From the point of view of different stages of XRJ production, whether from the cultivation stage of planting and harvest stage, the IoT technology can be used to improve its work efficiency and fine management.

• At the plant preparation stage, a bunch of sensors can be arranged in the greenhouse to analyze real-time soil information and choose the right date varieties.
• At the planting and cultivation stage of jujube, the IoT technology can be used to collect temperature and humidity information for efficient management, so as to cope with environmental changes and to ensure plant seedlings grow in the best environment.
• At the growth stage of jujube, the IoT can be used to monitor the environmental information, nutrient information, and crop diseases and insect pests in real time. Using relevant sensors are accurate and real-time access to soil moisture, environmental temperature and humidity, illumination, through real-time data monitoring and special varieties of expert experience, combining with control system regulating crop growth environment, improve crop nutrition state, crop pest and disease outbreaks in time period, to maintain the best crop growth conditions. It plays a very important role in the growth and management of jujube.
• At the harvest stage of jujube, the IoT technology is used to transfer information such as appearance, fruit diameter, and nutritional composition of jujube to the data center. According to the data of planting environment, weather condition, and fruit tree status, the planting scheme is optimized for next year through big data technology analysis.

**FIGURE 1:**
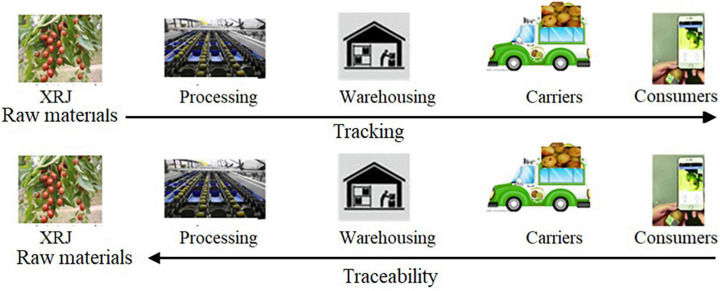
Tracking and traceability system of Xinzheng red jujube (XRJ).

Through the IoT technology, the production and operation of XRJ can be rapidly transformed from extensive and empirical management to fine and scientific management, so as to improve the yield and quality of XRJ, reduce the production cost of XRJ, and protect the agricultural environment. Figure 1 shows the flowchart of tracking and traceability system, where tracking is the ability to follow the path of a particular unit or batch of XRJ from upstream to downstream of the supply chain and traceability is the ability to identify the source of a specific unit or batch of XRJ products from the downstream to the upstream of the supply chain, i.e., the ability to trace the planting, production, and processing of a certain XRJ product through the method of traceability code.

**FIGURE 2:**
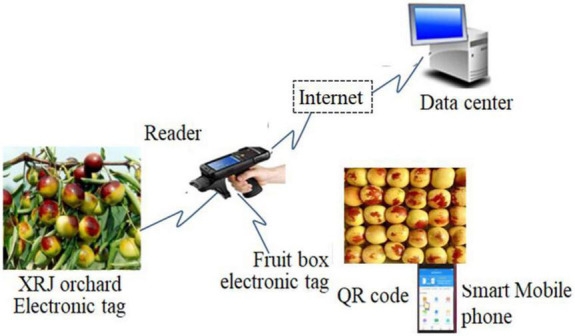
XRJ tracing query based on radiofrequency identification (RFID) + Quick Response (QR) code.

Data acquisition in Figure 1 is implemented by RFID + Quick Response (QR) code. Figure 2 shows the XRJ tracing query flowchart based on RFID + QR code.

### Blockchain

Blockchain is a chain of blocks, each containing a certain amount of information, connected in the chronological order of their creation (Konstantinidis et al., 2018). The chain is stored on all the servers and as long as one server in the system works, the whole blockchain is safe. These servers, called nodes in a blockchain system, provide storage space and computing power for the entire blockchain system. Tampering with information in a blockchain is extremely difficult because it requires the consent of more than half of the nodes and modification of all the nodes, which are usually in the hands of different parties. Compared with traditional networks, blockchain has two core features: data are hard to tamper with and it is decentralized. Based on these two characteristics, the information recorded by blockchain is more authentic and reliable, which can help to solve the problem of distrust among people. In the narrow sense, blockchain is a distributed ledger that cannot be tampered with or forged by means of cryptography. It is a chain data structure that combines data blocks sequentially according to time sequence. Generalized blockchain technology is the use of blockchain data validation and data storage structure and update the data generated by distributed node consensus algorithm, using the way to ensure the safety of data transmission and access of cryptography, the use of automated script code intelligent contracts, programming and operation data of new distributed infrastructure, and computing paradigm.

Blockchains allow people to have a distributed peer-to-peer network where non-trusting members can interact with each other without a trusted intermediary in a verifiable manner. Combining blockchain with the IoT, namely, blockchain-IoT is powerful and can cause significant transformations across several industries, paving the way for new business models and novel, distributed applications (Christidis and Devetsikiotis, 2016). It facilitates the sharing of services and resources leading to the creation of a marketplace of services between devices and allows people to automate in a cryptographically verifiable manner several existing, time-consuming workflows.

## Xinzheng Red Jujube Quality Traceability System

From the actual needs of the whole quality and safety management of XRJ, based on the quality and safety standards of XRJ, technical standards of XRJ, safety law, and other regulations and standards, the whole process risk management and traceability system framework of pollution-free XRJ quality and safety are designed. The wireless network remote environment monitoring technology is used to collect and analyze the data of XRJ in the whole circulation links such as XRJ, processing, storage, transportation, and sales, and generate reports with the results, so that managers can understand the situation in each link and solve related problems in time. All the monitoring report and pictures are numbered, using the information classification and coding standards, designing the pollution-free XRJ quality security coding, generating the QR code identification of XRJ, so that the consumers by smartphone scanning, back to the whole process of XRJ circulation. The whole process of pollution-free XRJ quality and safety can be monitored and traced.

The XRJ quality traceability system based on hybrid mode is mainly divided into hardware and software system. The hardware module mainly realizes the real-time data collection of crops from planting to processing, circulation and sales of XRJ, and writes the data into the database. The software system is divided into Web Service and Android client. Since the Android client cannot interact directly with the database, this document uses Web Service as a bridge. The Web Service retrieves data from the database and the Android client retrieves data from the Web Service through Simple Object Access Protocol (SOAP).

(1) Hardware environment design: The hardware part of the system mainly includes power supply module, sensor module, ZigBee module, and gateway module. The power supply module supplies power to other modules. The sensor module collects environmental data in real time and then transmits the data to the gateway module through ZigBee module. Finally, the gateway module uploads the data to the database through a specific serial port communication program.

(2) Software design: The gateway module of the hardware part writes the real-time data collected by the sensor into the database through serial communication. The Web Service obtains data from the database and the Android client obtains data from the Web Service through SOAP protocol. The Android client interacts with the database through Web Service, so as to realize the data query function.

**FIGURE 3:**
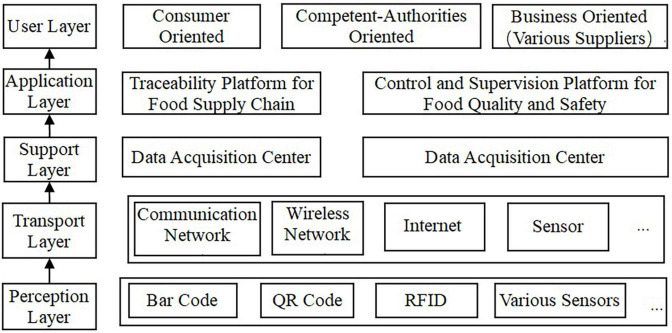
The architecture of XRJ traceability system.

**FIGURE 4:**
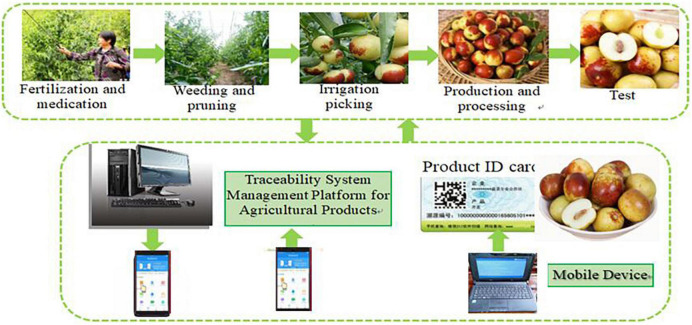
The flowchart of XRJ traceability system.

**FIGURE 5:**
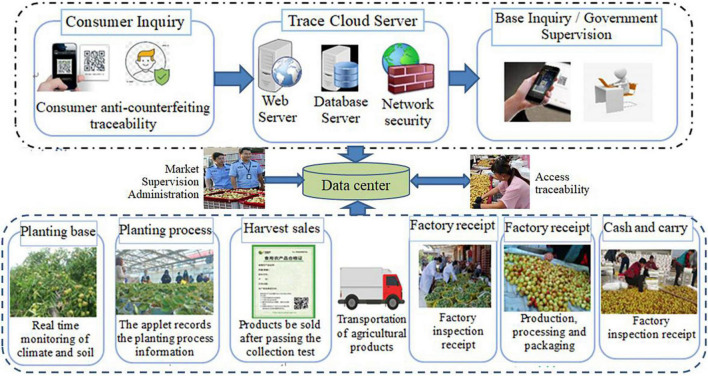
Data processing of XRJ traceability system.

**FIGURE 6:**
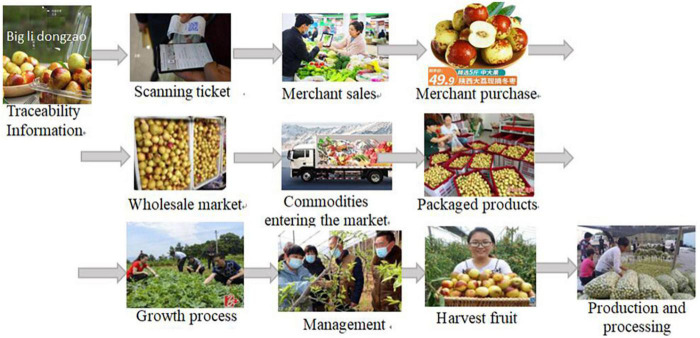
Implementation processing of XRJ traceability system.

The architecture and flowchart of traceability system of XRJ based on hybrid mode of blockchain and the IoT are shown in Figures 3, 4, respectively.Figure 5 shows the data processing process of the system based on hybrid mode of blockchain and the IoT, including network data process and business process. Figure 6 shows the implementation processing. Data can be stored in two ways: database and blockchain. The database is maintained by the Production and market supervision administration in the traceability system, while the blockchain is jointly maintained by jujube farmers, jujube gardens, industrial enterprises, commercial enterprises, and end consumers. Data entry, data verification, and data maintenance are carried out through simple operation. Traces the process in the design, the most will include red jujube cultivation in the process of planting, management, and collect key information such as input to the database and the most maintenance blockchain node, after the consensus of the processes in the system to write the key information into blocks, once written, this information is given by the blockchain traceability, difficult to tamper with the traits, such as in the information written after success, will get a block return value, the return value will be saved in the database maintained by the market supervision administration, is the key to realize consumers on the date planting time, location, and other key information query. Collectively, red jujube acquisition time can be purchase level key information into blocks, such as industrial enterprises processing time, production time, and so on key information can be written to block, the business enterprise can be dispatching information, storage time, put in the region during the key information such as writing block, get the corresponding return value, save in the administration of the market supervision, and maintenance of the database. The market supervision administration plays a supervisory role in the whole process of data entry, providing national supervision for consumers to purchase dates and processed products, and effectively protecting the rights and interests of consumers. As far as consumers are concerned, detailed information about dates and processed products provided by jujube farmers, jujube gardens, industrial enterprises, and commercial enterprises can be easily found through the quality traceability system. Its main operating process is: the consumer by scanning red jujube and its processed products of QR code on the package to the market supervision and administration of the maintenance of red jujube-related data in the database query, can detailed process of red date information query, if the query shows information does not accord with red jujube actual information, to a large extent can be concluded that the purchase of jujube is safe, consumer can undertake inform against to market supervisory bureau or commercial enterprise, obtain corresponding compensation.

## Experiments and Results

Choose the high-quality characteristics in XRJ varieties to quality and safety traceability system demonstration experiment, on the basis of sufficient study, analysis of demand, study and development to establish traceability anticounterfeiting XRJ whole-industry chain management system, unified data standard system construction, software and hardware, and the reliable technology are constructed to obtain integrity, practicality, advanced, and scalability. Anticollision method of RFID tag in large dynamic change and fast moving environment is carried out synchronously and is used in traceability anticounterfeiting management system to improve batch tag identification efficiency in complex environment. The advantages and characteristics of the XRJ whole-industry chain traceability anticounterfeiting management system based on radiofrequency identification technology will be gradually promoted to red jujube, planting, and sales enterprises in the province, benefiting the majority of villages and growers.

According to the distribution of relevant personnel in the enterprise, the application scheme is determined: (1) personnel in the headquarters. Due to data center server deployment in enterprise headquarters, system administrator in the enterprise headquarters, production management personnel, and technical personnel can switch to the traditional C/S architecture system client data reporting and can carry out data analysis and statistics, the traditional C/S architecture system data reporting and management of the purpose is to improve the response speed data, reduce the influence of Windows Communication Foundation (WCF) frame on system performance; (2) Personnel other than the headquarters. The staff in the base, processing plant, or outside can choose different system clients for data reporting and management according to whether the network is smooth. When the network is not smooth, people can choose the traditional C/S frame system client to input the local data into the local server and carry out the local data analysis and statistics. When the network is normal, people can use the WCF client to synchronize data from the local server to the data center at the headquarters. After the synchronization, the WCF client can directly report data to the data center at the headquarters in real time and analyze and collect statistics over the internet.

The Henan Xinzheng Xinxing Jujube Industry Corporation Ltd. is chosen to validate the system. The network infrastructure of the enterprise is better. The enterprise has a provincial planting base of red dates: Nankou red jujube production center and 9 large-scale planting bases. Some bases have poor network infrastructure and the network cannot be guaranteed to be smooth all the time. According to the network infrastructure of the enterprise, it is very suitable to use the agricultural production traceability management system based on hybrid architecture. At present, the client of the whole system has been deployed in three planting bases and the server has been installed in the corporate headquarters. The production management department is in urgent need of obtaining the real planting area, number of trees planted, amount of pesticides and fertilizer applied and their types, yield, and other data of each base. However, such data can only be collected after the production cycle is completed for a period of time. After adopting this system, the above data can be obtained on the same day. In addition, the following functions are also realized: (1) query the planting and production situation of different batches, different people, different bases, and different time periods; (2) Trace codes are formed according to relevant rules; (3) Query the quality management of the whole production process according to the traceability code; and (4) Upload enterprise traceability data to the ministerial traceability data center through the upload component. Figure 3 shows the main interface of XRJ production traceability management system. The upper part is divided into the system menu, the left part is divided into the navigation bar, and the right part is divided into the main part of the system, by clicking the specific items in the navigation bar and people can operate the corresponding record table on the right.

The blockchain-based agricultural product traceability system consists of six modules: source and origin, product, agricultural operation, quality inspection, warehousing and storage, and transportation. The data of agricultural operation, quality inspection, warehousing and storage, and transportation shall be stored in the blockchain after verification. We collectively refer to the data of these four modules as source information. The specific implementation of each functional module of the traceability system is demonstrated through sequence diagram analysis. The system development environment configuration is shown in Table 1. Hyperledger Fabric network deployment requires the use of Docker container because the Fabric’s billing nodes, sorting nodes, and smart contracts all run in Docker container. In system terminal sudo, add apt—repository “deb [arch = amd64] https://hyperledger-fabric.readthedocs.io/en/release-1.2/network/network.html stable” and Sudo apt-getinstall docker-ce docker-ce-cli container. IO installs the Docker container.After setting up the Fabric operating environment, the next step is to build the Fabric blockchain, consisting of the following major steps: generating certificates and keys, generating genesis blocks, creating channels, starting the Fabric network, nodes joining channels, and installing and instantiating smart contracts.

**TABLE 1:** System development environment configuration.

Development environment	Tool and version number
The operating system	Ubuntu 16.04
Blockchain deployment container	Docker 19.03.9
Blockchain development tools	Hyperledger Fabric 1.4.0°
Hyperledger Fabric SDK Java 1.4.0	
The database	MySQL 5.7
System development language	Java1.8.0261
System backend development framework	Springboot 2.1.4, MyBatis 2.0.1
System front-end development framework	Vue.js2.0, Uniapp

In the development stage of the system, white-box test is applied to simulate all the kinds of possibilities inside the program to improve the robustness of the processing method. In the test stage, black-box test is used to test each functional module by simulating user behavior. The test results of each module of the system are shown in Table 2. Each functional module of the system is tested by building a Fabric blockchain and deploying smart contracts. As shown in Table 2, the system completes the development and implementation of specific functions to ensure the reliability of the system.

**TABLE 2:** Test results of each module of the system.

Test module	Function	Input data	Expected result	Test result
Farming operations Quality inspection Warehouse storage Transport	Farming Operations Quality Inspection Data Storage Repository Data Shipping data	Agr. operation data Quality check data storage repository data Transportation data	Save success Save success Save success Save success	Normal Normal Normal Normal
Data auditing	Farm process audit Warehouse storage audit Quality inspection audit Transport data audit	Farm operation ID Storage ID Quality test ID Transport data ID	The audit is successful and the data	Normal Normal Normal Normal
Traceability data tamper verification	Clothing operation tamper effect test Tamper check is stored in the library Quality test tamper check Transportation data tamper check	Farm operation ID Storage ID Quality test ID Transport data ID	If not tampered, the content not displayed. Otherwise, the data comparison between the database and the blockchain is displayed	Normal Normal Normal Normal
	Data reduction	Farm operation ID Quality test ID Storage ID Transport data ID	Data restoration succeeds	Normal
Product management	New product Edit product Remove product Query products by terms Traceability queries	Product information Product information Product ID Query conditions Product Batch No.	New success Modify success Delete success Query products meet conditions Data of origin, product, agricultural operation, warehousing, storage and transportation of this batch of products are queried from the blockchain	Normal Normal Normal Normal Normal

## Conclusion

With the rapid development of economic level, some agricultural product enterprises reduce production costs to maximize the profits of some agricultural enterprises and result in a lot of agricultural product safety incidents in recent years. Therefore, people have to pay more attention to food safety in daily life. This article constructs a set of XRJ quality traceability system and carries on the system analysis and application verification. The hybrid model in the system makes use of its unique time stamp, consensus mechanism, and other technical means to realize the data of jujube planting, jujube purchasing, jujube processing, jujube sales, and other data that cannot be tampered and traceable. At the same time, the market supervision bureau and consumers are included in the date quality traceability supervision system, which breaks the information island of traditional traceability, provides information support, realizes the full transparency of production and sales process to a certain extent, provides technical support for good market operation order, and provides quality guarantee for consumers’ life and health. With in-depth study and development of blockchain technology, coupled with the requirements of high-quality development of the date industry, blockchain technology will provide new solutions to date traceability, pesticide residues, and brand counterfeiting. While establishing XRJ safety supervision and supervision mechanism, relevant departments need to establish traceability XRJ system, implement relevant responsibility to individuals, and strengthen food production supervision at the source.

## Data Availability Statement

The raw data supporting the conclusions of this article will be made available by the authors, without undue reservation.

## Ethics Statement

Written informed consent was obtained from the individual(s) for the publication of any identifiable images or data included in this article.

## Author Contributions

RJ contributed to the model construction and manuscript writing. PL contributed to the data acquisition and experimental verification.

## Conflict of Interest

The authors declare that the research was conducted in the absence of any commercial or financial relationships that could be construed as a potential conflict of interest.

## Publisher’s Note

All claims expressed in this article are solely those of the authors and do not necessarily represent those of their affiliated organizations, or those of the publisher, the editors and the reviewers. Any product that may be evaluated in this article, or claim that may be made by its manufacturer, is not guaranteed or endorsed by the publisher.
